# White Matter Measures and Cognition in Schizophrenia

**DOI:** 10.3389/fpsyt.2020.00603

**Published:** 2020-07-10

**Authors:** Cemre Erkol, Talia Cohen, Virginie-Anne Chouinard, Kathryn Eve Lewandowski, Fei Du, Dost Öngür

**Affiliations:** ^1^ Psychotic Disorders Division, McLean Hospital, Belmont, MA, United States; ^2^ Department of Psychiatry, Harvard Medical School, Boston, MA, United States

**Keywords:** MTR = magnetization transfer ratio, diffusion, NAA (N-acetyl aspartate), cognition, Stroop, schizophrenia

## Abstract

White matter (WM) abnormalities are commonly reported in schizophrenia but whether these arise from the axon or myelin compartments or both is not known. In addition, the relationship between WM abnormalities and cognitive function is not fully explored in this condition. We recruited 39 individuals with schizophrenia spectrum disorders and 37 healthy comparison subjects. All participants underwent MRI scanning at 4 Tesla to collect data in the prefrontal white matter on magnetization transfer ratio (MTR) and diffusion tensor spectroscopy (DTS) which provide information on myelin and axon compartments, respectively. We also collected Matrics Composite Cognitive Battery (MCCB) and Stroop cognitive data. We found an elevated N-acetylaspartate (NAA) apparent diffusion coefficient in schizophrenia in this cohort as in our previous work; we also observed poorer performance on both the MCCB composite and the Stroop in schizophrenia patients compared to controls. The MTR measure was correlated with the MCCB composite (r = 0.363, *p =* 0.032) and Stroop scores (r = 0.387, *p =* 0.029) in healthy individuals but not in schizophrenia. Since this is the first exploration of the relationship between these WM and cognitive measures, we consider our analyses exploratory and did not adjust for multiple comparisons; the findings are not statistically significant if adjusted for multiple comparisons. These findings indicate that WM integrity is associated with cognitive function in healthy individuals but this relationship breaks down in patients with schizophrenia.

## Introduction

Several lines of evidence implicate white matter (WM) abnormalities in the pathophysiology of schizophrenia (SZ). WM abnormalities are related to psychotic symptoms (1) and poor cognitive functioning (2, 3). Diffusion tensor imaging (DTI) is an MRI modality which measures *in vivo* water molecule diffusion and provides noninvasive information on integrity of the myelinated axon pathways within WM. DTI has been successfully used for many years to demonstrate WM abnormalities in several brain disorders including schizophrenia (4), attention deficit hyperactivity disorder (5), and multiple sclerosis (6).

Although DTI research has provided significant insights into WM abnormalities in brain disorders such as schizophrenia, it cannot currently resolve signals from axon and myelin compartments since water molecules exist in both intracellular and extracellular spaces and there is exchange between these compartments. To provide compartment-specific information on WM integrity in the human brain, we recently implemented Magnetization Transfer Ratio (MTR) and Diffusion Tensor Spectroscopy (DTS) approaches (7), and we reported abnormalities in schizophrenia (8) and bipolar disorder (9) using these techniques. MTR is an MRI modality that provides information on brain myelin content. DTS is an MRI technique which measures diffusion of metabolites such as N-acetylaspartate (NAA). NAA is only intraneuronal (10) and it has been proposed that NAA diffusion can help us understand microstructure within neurons (11).

In the current study, we collected MTR/DTS measures in prefrontal white matter along with cognitive measures in a group of patients with schizophrenia (SZ) and matched healthy individuals. Cognitive assessment included the MATRICS Consensus Cognitive Battery (MCCB) and The Stroop Color and Word Test (Stroop). The MCCB measures cognition broadly across several neurocognitive domains and yields domain scores and a global cognitive composite (12), while the Stroop probes prepotent response suppression and is dependent on prefrontal cortex function (13). Using these data, we sought to test the relationship between white matter abnormalities and abnormal information processing in SZ. We hypothesized that we would replicate our previous findings of abnormal white matter measurements in this condition (8), while also replicating the widely reported deficits in cognitive function in the SZ group (14–17). We further hypothesized that imaging measures collected in the prefrontal cortex (PFC) would be significantly correlated with performance in the prefrontal-dependent Stroop test. On the other hand, we hypothesized that we would not see a correlation between imaging measures which are prefrontal-specific and the MCCB composite score which integrates the function of multiple brain regions including parietal, temporal, and occipital as well as prefrontal.

## Materials and Methods

### Participants

We recruited 39 subjects with Schizophrenia/Schizoaffective Disorder (SZ) and 37 healthy controls (HC) aged 18 to 49 years old. Data were collected between 2012 and 2015. Diagnosis was determined using the SCID-IV diagnostic interview. Inclusion criteria were meeting criteria for schizophrenia/schizoaffective disorder for the SZ group, and having no history of medical, neurological, or psychiatric disorders and taking no medications for the HC group. We excluded subjects with any lifetime diagnosis of substance or alcohol use disorders but tobacco use was allowed. We also excluded those who could not tolerate or had contraindications to the scanning environment. All study procedures were approved by the Partners Institutional Review Board which oversees research at McLean Hospital.

### Methods

#### Cognitive and Clinical Assessment

MATRICS Consensus Cognitive Battery: The MATRICS Consensus Cognitive Battery (18) includes 10 tasks across seven domains. Domains and tasks include processing speed (Brief Assessment of Cognition in Schizophrenia Symbol Coding, Animal Fluency, Trails A), attention (Continuous Performance Test), working memory (WMSIII Spatial Span, Letter-Number Span), verbal learning (Hopkins Verbal Learning Test-Revised), visual learning (Brief Visuospatial Memory Test-Revised), problem-solving (Neuropsychological Assessment Battery), and social cognition (Mayer-Salovey-Caruso Emotional Intelligence Test). Age and gender-normed scores (T scores) are generated for each cognitive domain and the global cognitive composite.

The Stroop Color and Word Test (SCWT; Stroop 1935) assesses the ability to inhibit cognitive interference (13). The primary Stroop outcome measure in the present project was from the “interference” portion of the task, in which subjects are provided with color words and must name the color of the ink, inhibiting the primary response to name the written word. Scores reflect the number of correct responses in 45 s.

Positive and Negative Syndrome Scale (19) measures psychopathology in 30 items rated 1 to 7 and is divided into three subscales (Positive, Negative, and General Psychopathology).

#### Brain Measures

Following a two-pronged approach to probe white matter abnormalities detailed in our previous work (7) we collected data on both MTR and DTS, which allow us to distinguish between white matter abnormalities in brain myelination and axonal geometry, respectively.

##### MTR

**Figure 1:**
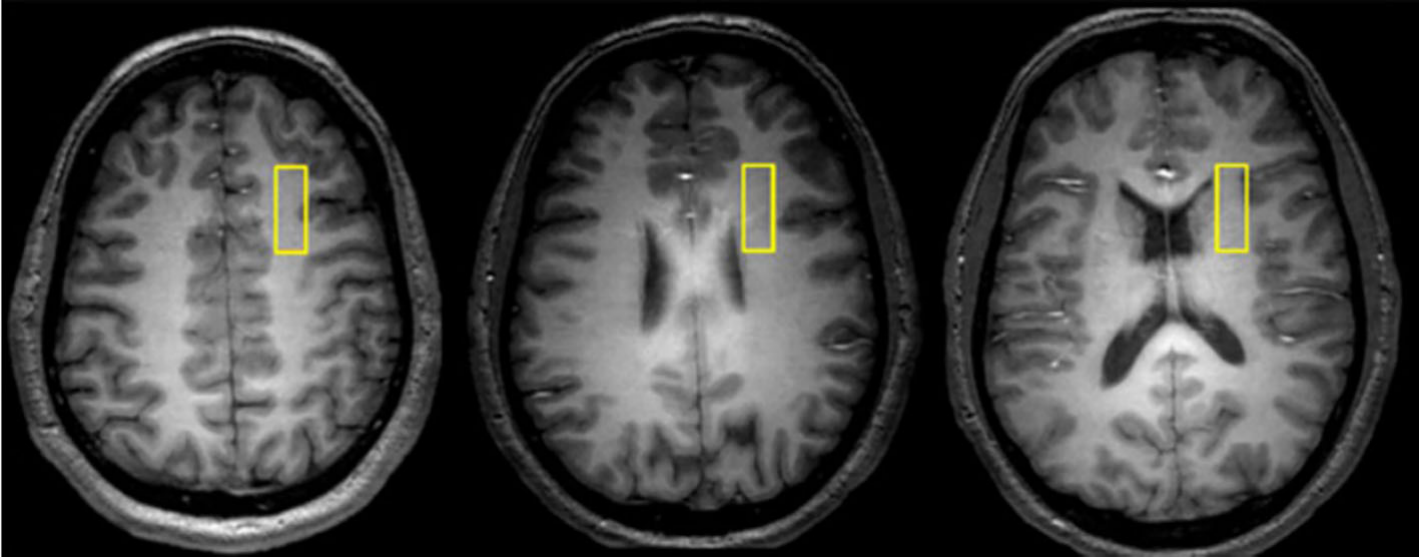
Representative axial images depicting the location of 1 × 3 × 3 cm white matter voxel in the right prefrontal cortex.

Data were collected from a 1 × 3 × 3-cm voxel within the right prefrontal cortex WM (**Figure 1**) at 4 Tesla (Varian/UnityInova, Varian, Palo Alto, CA, USA). We chose a single voxel approach to measure MTR because we aimed to examine MTR and DTS data from the same voxel. The voxel was positioned in WM (confirmed by tissue segmentation), anchored by adjacent gray matter in anterior and lateral directions to ensure comparable locations across scans. Our goal was to study signal from only WM and we selected a 9 cc voxel as this was the largest size we could position completely within WM. Obtaining two voxels would have prolonged scan time to unacceptable levels so we decided to collect data only in one hemisphere. DTI abnormalities in schizophrenia do not have hemispheric predilection (20) and we selected the right hemisphere to avoid language-related variability in the left hemisphere.

We used a B1-insensitive saturation pulse train (21) applied at the beginning of a standard point-resolved spectroscopy (PRESS) sequence to saturate “bound-water” signal with a specific frequency offset (21, 22). Data were obtained in 50-Hz steps at a range of frequencies offset 400–1,000 Hz in either direction from the water signal, and a single MTR number was calculated by averaging across frequencies. Saturation time (t_sat_) was 2.6 s with repetition time/echo time of 3,000/30 ms and two repetitions.

##### DTS

The standard PRESS sequence was modified by incorporating diffusion gradients for DTS measurements. Bipolar diffusion gradients with six directions and one control (totaling seven spectra) were applied to calculate diffusion tensors of signal from water and metabolites. The applied b value was 1,412 s/mm^2^, calibrated by a phantom with water apparent diffusion coefficient (ADC) assumed to be 2.1 × 10^−3^ mm^2^/s at room temperature (23). Repetition time/echo time was 3,000/135 ms and diffusion time (D_t_) was 60 ms. There were 96 repetitions for metabolites and 4 repetitions for water measurements. Metabolite spectra were acquired with water saturation with VAPOR (24). Each individual free induction decay (FID) was digitized before summing all FIDs.

#### MRI and MRS Data Processing/Analysis

All data-processing used our previously reported methods (8). Briefly, all MRI/MRS data were processed by an MR physicist (FD) who was blind to diagnosis. Post-processing of the FIDs including apodization, Fourier transformation, frequency, and phase correction as well as calculation of MTR and DTS constants was carried out using software available in the Varian Console and home-grown software running on MATLAB. As MTR and DTS measurement both involve relative signal change, we digitized the water or NAA signal and normalized it to baseline. Reported units for ADC are in mm^2^/s × 10^−3^.

#### Statistical Approach

All analyses were performed using SPSS V.21. We conducted independent samples t-tests and chi-square tests to compare the HC and SZ groups. We used a significance threshold of p < 0.05 for all analyses. Because our cognitive and imaging analyses were exploratory, we did not correct for multiple comparisons in statistical testing. We did not conduct an *a priori* power analysis for our study.

## Results

Demographic and clinical data are presented in **Table 1**. The SZ group had significantly lower educational attainment than the HC group but the groups were otherwise well-matched. As expected, the SZ group obtained significantly lower scores on both cognitive functioning tests compared to the HC group. Results are summarized in **Table 2**.

**Table 1:** Demographic and Clinical Characteristics of Study Participants.

	Patient (n=39)	Healthy Control (n=37)	Statistical Evaluation
Age	33.4 (8.6)	31.3 (9.2)	t(74) = −1.04, *p =* 0.30
Gender (% Female)	%28.2	%40.5	*χ*2 = 1.28, *p =* 0.26
Education	13.8 (2.2)	16.3 (1.9)	t(73) = 5.22, *p =* 0.2E–5
Ethnicity	38 NON-HL,1 HL	32 NON-HL, 5HL	*χ*2 = 3.1, *p =* 0.07
Race (% Caucasian)	%66.6	%72.9	*χ*2 = 0.36, *p* = 0.55
PANSS	52.8 (18.1)	—	
Total Score			
CPZ	402.3 (295.6)	—	

PANSS, Positive and Negative Syndrome Scale; CPZ, Chlorpromazine; HL, Hispanic/Latino.

**Table 2:** MTR, DTS, and Neurocognitive Tests Data Summary.

	Patient (n=39)	Healthy Control (n=37)	Statistical Evaluation
MTR	0.154 (0.02)	0.152 (0.02)	t(69) = −.36, *p* = 0.72
NAA ADC	2.7E–04 (5.8E–05)	2.4E–04 (5.1E–05)	t(69) = 2.11, *p* = 0.038
MATRICS	39.1(13.1)	54.1 (10.8)	t(74) = 5.43, *p* = 0.1E–5
Composite Stroop CW	40.5 (10.8)	49.9 (9.7)	t(68) = 3.8, *p* = 3.1E–4

MTR, magnetization transfer ratio; NAA ADC, *N*-acetylaspartate apparent diffusion coefficient.

We were able to partially replicate our previous imaging results. Specifically, we found an elevation in NAA ADC in the SZ group compared with the HC group in the PFC voxel under study (t (69) = −2.1, p = 0.038). On the other hand, we did not observe a difference between the SZ and HC groups on the MTR measure collected in the same PFC voxel (t(69) = −0.357), p = 0.722). We found poorer performance on both the MCCB composite and the Stroop in SZ patients compared to controls (**Table 2**).

We also conducted a series of exploratory correlational analyses to examine if there were any relationships between the MRI/MRS measures and the cognitive tests. Results showed two moderate correlations in the HC group. The first was between the MTR and MCCB composite scores (r = 0.363, *p =* 0.032), and the second between the MTR and Stroop scores (r = 0.387, *p =* 0.029). These findings are no longer statistically significant when adjusted for eight multiple comparisons (two groups, two MRI measures, two cognitive measures). There were no correlations between MTR and cognitive measures in the SZ group, or between NAA ADC and cognitive measures in either group. More detail on these correlation analyses are reported in **Table 3**.

**Table 3:** Correlations Between MTR, DTS, and Neurocognitive Tests.

	MTR	NAA ADC	MATRICS Composite	Stroop CW
**SZ**				
MTR	1	.172	0.267	−.116
NAA ADC	.172	1	−.160	−.031
MATRICS	.267	-.160	1	.647**
Composite				
Stroop CW	−.116	−.031	.647**	1
**HC**				
MTR	1	−.209	.363*	.387*
NAA ADC	−.209	1	.067	0.269
MATRICS Composite	.363*	.067	1	.460**
Stroop CW	.387*	.269	.460**	1

MTR, magnetization transfer ratio; NAA ADC, N-acetylaspartate apparent diffusion coefficient.

*p < .05.

**p < .01.

## Discussion

In this study, we used MTR and DTS to quantify white matter abnormalities in a group of SZ patients and matched healthy controls and we correlated these results with performance in neurocognitive tests. We found reduced cognitive scores based on the MCCB composite and the Stroop test in people with SZ, as well as significantly elevated NAA ADC. The NAA ADC result is similar to that in our previous study with SZ (8) and different from bipolar disorder (9). In a recent study in first episode psychosis, we also found elevated NAA ADC in patients compared to healthy controls, but this elevation was not statistically significant (25). On the other hand, we did not find the predicted reduction in MTR in SZ in the current study. Some possible reasons for this result include potential drift in MRI scan parameters (since scans are collected over the course of 3 years and MTR is a relative measure that would be affected by scanner drift) and heterogeneity in antipsychotic medication use or other disease and treatment parameters in this SZ group compared to others. In addition, our SZ group includes individuals with schizoaffective disorder and we cannot completely rule out contribution from abnormal affective processing in addition to psychosis in this sample.

The correlation between MTR and cognitive measures in healthy people but not in SZ patients indicates a relationship between myelination and neurocognition in physiological conditions that is disrupted in pathology. In other words, greater myelination may support improved brain function in healthy people but in conditions where brain function has become abnormal this impact of myelination on cognitive performance is no longer evident (26). Interestingly, we did not find evidence that this relationship is restricted to prefrontally-dependent neurocognition in healthy people. Our WM measures were obtained in the prefrontal white matter, but they correlate with the MCCB composite score as well as a prefrontal-dependent measure of cognition. This observation may suggest that our prefrontal WM measures reflect the state of myelination in broader WM pathways, or that myelination of specific neural pathways in the prefrontal cortex makes a distributed impact on brain function. The absence of such relationships between NAA ADC and neurocognition suggests that axonal geometry as reflected by NAA ADC is not directly related to cognitive parameters in this study.

Note that our findings should be considered exploratory and to be replicated in future research; the current results are no longer statistically significant when adjusted for multiple comparisons. To further examine the relationship between myelination and cognitive function, future studies using larger and well-matched samples will be needed. We propose that novel approaches to dissecting myelin *vs.* axon based probes of WM integrity provide deeper insights than those obtained by DTI studies alone.

## Data Availability Statement

The raw data supporting the conclusions of this article will be made available by the authors, without undue reservation.

## Ethics Statement

The studies involving human participants were reviewed and approved by Partners IRB. The patients/participants provided their written informed consent to participate in this study.

## Author Contributions

CE: conducted analyses, wrote the manuscript. TC: conducted analyses, wrote the manuscript. EL: collected data, edited the manuscript. FD: collected data, edited the manuscript. DÖ: conceived the project, obtained funding, oversaw research and writing. V-AC: conducted analyses, edited the manuscript.

## Funding

This study supported by grants R01MH094594 and K24MH104449 to DÖ.

## Conflict of Interest

The authors declare that the research was conducted in the absence of any commercial or financial relationships that could be construed as a potential conflict of interest.
